# Special Issue “Bifidobacteria: Insights from Ecology to Genomics of a Key Microbial Group of the Mammalian Gut Microbiota”

**DOI:** 10.3390/microorganisms8111660

**Published:** 2020-10-27

**Authors:** Marco Ventura, Francesca Turroni, Christian Milani, Jennifer Mahony, Francesca Bottacini, Douwe van Sinderen

**Affiliations:** 1 Laboratory of Probiogenomics, Department of Chemistry, Life Sciences, and Environmental Sustainability, University of Parma, 43124 Parma, Italy; francesca.turroni@unipr.it (F.T.); christian.milani@unipr.it (C.M.); 2 Interdepartmental Research Centre “Microbiome Research Hub”, University of Parma, 43124 Parma, Italy; 3 APC Microbiome Ireland and School of Microbiology, Bioscience Institute, National University of Ireland, Cork, Ireland; J.Mahony@ucc.ie (J.M.); francesca.bottacini@ucc.ie (F.B.); d.vansinderen@ucc.ie (D.v.S.)

In recent years, substantial efforts have been made to dissect the composition of microbial communities that are present in the human gut, and to investigate their interactions with their host. From this perspective, the occurrence of members of the genus *Bifidobacterium* within the human gut microbiota has been linked to a positive host health status. Bifidobacteria are amongst the first colonizers of the human gut, in part due to demonstrated vertical transmission from mother to newborn. Colonization and establishment of these bacteria in the human gut is modulated by specific human milk constituents according to a fascinating mechanism of microbe–host co-evolution. It has been shown that certain bifidobacterial genomes have evolved to acquire specific genes to allow the utilization of particular carbohydrate components of this human secretion. The biology of bifidobacteria has been extensively exploited and several of their saccharolytic features are well characterized. Moreover, several studies have underlined the complicated relationships of bifidobacteria with their human host, as well as with other members of the gut microbiota. Remarkably, the ecological role of bifidobacteria as part of the human gut microbiota has been investigated in terms of shaping the gut microbiota and re-establishment of microbiota homeostasis through cross-feeding activities.

This current Special Issue gathers 13 articles covering various ecological, metabolic and genetic aspects pertinent to the biology of bifidobacteria and their associated impact on human health.

These include comprehensive overviews on bifidobacterial establishment in the human gut [1,2] and on the manner by which breastfeeding sustains bifidobacterial colonization during infancy [3]. In addition, three articles propose new methodological metagenomics-based approaches to delineate the composition of bifidobacterial communities in the human gut [4,5] or in a food setting [6], while another covers their functional behavior under in vivo conditions [7]. The genomic characteristics of the *Bifidobacterium* genus [2,8,9], as well as their metabolic features, are discussed in intricate detail [2,7,10,11] in this Special Issue. One article is dedicated to the resistome of bifidobacteria, and to mechanisms involved in the acquisition/transmission of antibiotic resistance [12,13]. Collectively, these articles stress the importance of bifidobacterial interactions in influencing health in humans. Ultimately, enhancing molecular knowledge in the field of bifidobacterial research is warranted to develop novel, science-based probiotic products that contain bifidobacterial strains to improve and/or support human health.
